# Activity of endovesical gemcitabine in BCG-refractory bladder cancer patients: a translational study

**DOI:** 10.1038/sj.bjc.6604074

**Published:** 2007-11-06

**Authors:** R Gunelli, E Bercovich, O Nanni, M Ballardini, G L Frassineti, N Giovannini, M Fiori, E Pasquini, P Ulivi, G L Pappagallo, R Silvestrini, W Zoli

**Affiliations:** 1 Department of Urology, Morgagni-Pierantoni Hospital, Forlì, Italy; 2 Istituto Scientifico Romagnolo per lo Studio e la Cura dei Tumori, Meldola (FC), Italy; 3 Department of Oncology, Cervesi Hospital, Cattolica (RN), Italy; 4 Department of Oncology, PF Calvi Hospital, Noale, Italy

**Keywords:** endovesical instillation, bladder cancer, gemcitabine, BCG-refractory

## Abstract

Intravesical gemcitabine (Gem) has shown promising activity against transitional cell carcinomas (TCC) of the bladder, with moderate urinary toxicity and low systemic absorption. The present phase II study evaluated the activity of biweekly intravesical treatment with Gem using a scheme directly derived from *in vitro* preclinical studies. Patients with Bacille Calmette-Guérin (BCG) -refractory Ta G3, T1 G1-3 TCC underwent transurethral bladder resection and then intravesical instillation with 2000 mg Gem diluted in 50 ml saline solution on days 1 and 3 for 6 consecutive weeks. Thirty-eight (95%) of the 40 patients showed persistent negative post-treatment cystoscopy and cytology 6 months after Gem treatment, while the remaining 2 patients relapsed at 5 and 6 months. At a median follow-up of 28 months, recurrences had occurred in 14 patients. Among these, four had downstaged (T) disease, three had a lower grade (G) lesion and three had a reduction in both T and G. Urinary and systemic toxicity was very low, with no alterations in biochemical profiles. In conclusion, biweekly instillation of Gem proved active in BCG-refractory Ta G3, T1 G1-3 TCC. Our results highlight the importance of preclinical studies using *in vitro* systems that adequately reproduce the conditions of intravesical clinical treatment to define the best therapeutic schedule.

Superficial transitional cell carcinomas (TCC) represent about two-thirds of all bladder cancers at first presentation and are a heterogeneous population of tumours that do not invade the *muscularis propria*. The European Organization for Research and Treatment of Cancer and the European Association of Urology Guidelines have identified three risk categories of TCC (low, intermediate and high) (Oosterlinck *et al*, 2006).

Complete endoscopic eradication followed or not by a single intravesical instillation with various drugs is the standard therapy for patients with low-risk tumours, while drug instillation and/or Bacille Calmette-Guérin (BCG) treatment is reserved for patients with intermediate-risk tumours in an attempt to reduce or delay recurrence and progression. For patients with high-risk tumours, BCG is considered the most effective conservative treatment, and radical cystectomy is the current option for cases refractory to this immunological therapy (Sylvester *et al*, 2002; Oosterlinck *et al*, 2006).

No one drug has proven superior in terms of efficacy. Mitomycin, epidoxorubicin and doxorubicin have all shown to have beneficial effects, but the optimal instillation scheme remains to be defined (Malmstrom *et al*, 1999; Malmstrom, 2000). Similarly, the best treatment for patients who fail to respond to adjuvant intravesical treatment needs further investigation, but cytotoxic drugs could represent an interesting alternative to standard BCG therapy. In this setting, Gemcitabine (Gem), a new generation deoxycytidine analogue, given systemically as a single agent (Lorusso *et al*, 1998) or in combination with cisplatin, carboplatin or taxanes (Li *et al*, 2005; Von Der Maase *et al*, 2005), has proven effective against metastatic bladder lesions, yielding 27–38% response rates in different studies. Furthermore, pharmacokinetic studies carried out after intravesical instillation have shown a very low systemic absorption of Gem, indicating this drug as an ideal candidate for intravesical therapy (Cozzi *et al*, 1999; Laufer *et al*, 2003).

The aim of this phase II study was to analyse the activity of the Gem treatment scheme that proved to be the most effective in *in vitro* cell cultures, a system that adequately reproduces the conditions of intravesical clinical treatment. The study was performed on patients refractory to BCG and submitted to transurethral bladder resection (TURB). Secondary end points were time to recurrence, progression and overall tolerability.

## MATERIALS AND METHODS

### *In vitro* studies

#### Cell cultures

The study was performed on two established bladder cancer cell lines: a commercial cell line (HT1376) with a 37-h doubling time (obtained from American Type Culture Collection, Rockville, MD, USA) and a cell line (MCR) established in the Biological Laboratory of the Department of Medical Oncology in Forlì (Zoli *et al*, 2004), with a 48-h doubling time. Cells were maintained as a monolayer in culture medium (DMEM) supplemented with 10% FCS, 100 IU ml^−1^ penicillin, 100 mg ml^−1^ streptomycin and 2 mM L-glutamine and subcultured weekly. All the experiments were performed during exponential cell growth.

#### Growth inhibition assay

Cells were seeded in 96-well, flat-bottomed microtitre plates at a density of 10 000 cells well^−1^. At 18–24 h after plating, 100 *μ*l of culture medium with or without Gem (kindly supplied by Ely Lilly, Florence, Italy) were added to each well. At the end of drug exposure, cells were fixed with 50% trichloroacetic acid and stained with 0.4% sulphorhodamine B (Sigma-Aldrich, St Louis, MO, USA), dissolved in 1% acetic acid (100 *μ*l well^−1^), and washed with 1% acetic acid to remove unbound stain. Protein-bound stain was solubilised with 100 *μ*l of 10 mM unbuffered Tris base, and cell density was determined using a fluorescence plate reader (wavelength, 540 or 510 nm). The sulphorhodamine B assay was used according to the method of Skehan *et al* (1990). Cells were exposed to Gem for one or two 1-h periods, the latter spaced out by a 24- or 48-h culture in drug-free medium (wash-out). Cells were treated with 1, 2 or 3 *μ*g ml^−1^ of Gem, taking into account that the peak plasma level for the drug is 3.2 *μ*g ml^−1^ (Abbruzzese *et al*, 1991). Control samples were processed in the same way as treated samples but in drug-free medium. Samples were run in octuplet, and each experiment was repeated three times.

#### Flow cytometry

At various observation times, the medium was removed and cells were detached from the flasks by trypsin, washed twice with PBS and stained according to the methods specified below. A FACS Vantage flow cytometer (Becton Dickinson, San Diego, CA, USA), equipped with an argon laser (488 nm), was used. Data acquisition and analysis were performed using CELLQuest Pro software (Becton Dickinson, San Diego, CA, USA) and ModFit 2.0 (DNA Modelling System, Verity Software House, Inc., Topsham, ME, USA). Samples were run in triplicate and each experiment was repeated three times. Standard errors were below 5%.

#### Cell cycle perturbations

Briefly, samples of 2 × 10^5^ cells were exposed to a 0.1 *μ*g ml^−1^. concentration of Gem for 1 h. Cell cycle distribution was determined immediately after treatment and 24 or 48 h after drug removal. Control samples were processed in the same way as treated samples but in drug-free medium. At the end of drug exposure, cells were harvested and stained in a solution containing RNase (10 Kunits ml^−1^; Sigma-Aldrich), NP40 (0.01%; Sigma-Aldrich) and propidium iodide (PI) (1 *μ*g ml^−1^; Sigma-Aldrich). After 30–60 min, samples were analysed and expressed as fractions of cells in the different cell cycle phases. Samples were run in triplicate and 10 000 events were collected for each replica. Data reported are the average of three experiments, with errors under 5%.

#### Apoptosis

Annexin-V assay: After Gem exposure, cells were harvested, washed once in PBS and incubated with 25 *μ*l ml^−1^ of Annexin V-FITC in binding buffer (Bender MedSystems, Vienna, Austria) for 15 min at 37 °C in a humidified atmosphere in the dark. Cells were then washed again in PBS and suspended in binding buffer. Immediately before flow cytometry analysis, PI was added to a final concentration of 5 *μ*g ml^−1^ to distinguish between total apoptotic cells (Ann-V+ and PI− or +) and necrotic cells (Ann-V– and PI+). For each sample, 15 000 events were recorded.

Data analysis: The efficacy of the two Gem exposures according to the different treatment schedules was determined by the *R* Index (RI) method (Romanelli *et al*, 1998). Although several methods have been proposed to evaluate the interaction between drugs, as critically analysed by Zoli *et al*. (2001), most of these are not applicable to drugs with a low cytotoxic effect. We therefore used Kern's method (Kern *et al*, 1988), modified by Romanelli (Romanelli *et al*, 1998), to overcome this problem. In brief, the expected cell growth (Sexp), defined as the product of the cell growth observed after exposure to drug A and that observed after drug B, and the observed cell growth (Sobs) after the exposure to the combination of A and B were used to construct an index (RI): *R*=Sexp/Sobs. An RI=1 indicated an additive effect. RI values higher or lower than 1 indicated synergistic and antagonistic effects, respectively.

### *In vivo* studies

#### Inclusion criteria

Patients who had disease recurrence (Ta G3, T1 G1-3 TCC) within 6 months of one induction cycle and at least three maintenance cycles of BCG, with no residual disease after TURB, were consecutively enrolled onto this phase II study. Age⩾18 years and WHO performance status (PS) 0–1. Normal upper urinary tract and bladder capacity>300 ml were documented before recruitment with Uro-CT scan and ultrasonography, respectively. Tumour stage was defined according to the 1997 TNM system (Sobin and Wittekind, 1997) and grading was based on the 1999 WHO classification (Epstein *et al*, 1998).

#### Exclusion criteria

Previous partial cystectomy, prior pelvic irradiation and clinical evidence of other malignancies or histologically confirmed carcinoma *in situ.*

Informed consent was obtained before treatment, and patients were required to be accessible for follow-up. The study protocol was approved by the Local Ethics Committee of the participating centres.

#### Therapeutic and evaluation protocol

Two weeks after TURB, the bladder was completely emptied by catheterisation and endovesical instillation was performed with 2000 mg Gem diluted in 50 ml saline solution, without pH adjustment. The drug was maintained in the bladder for at least 1 h and then spontaneously eliminated. The instillation was repeated on days 1 and 3 for the six consecutive weeks. No maintenance treatment was planned.

Patients were evaluated for response at the end of treatment and those with negative cystoscopy and cytology underwent close surveillance. Cytological analysis of voided urine and cystoscopy were performed at 3-month intervals for the first year, and every 6 months thereafter. In the event of positive cystoscopy, the lesion was submitted to histological examination. Patients with residual disease discontinued treatment and the subsequent therapeutic approach was defined at the discretion of each patient's physician.

The pre-study clinical evaluation comprised medical history, general physical examination, ECG, Uro-CT scan, chest X-ray and haematological evaluation (including WBC-PLT count, electrolytes and liver and kidney function), and ultrasonography evaluation of bladder capacity. Information on the date of treatment administration, drug doses and chemotherapy-induced side effects was recorded.

Toxicity and complete blood count were evaluated according to NCI Common Toxicity Criteria (version 2.0) on the first day of each cycle of therapy. A clinical, haematological and biochemical assessment for each patient was performed every third week and repeated at the end of treatment.

Gem dose was reduced to 1400 mg in the presence of grade 3 urgency or dysuria in association with grade 3–4 haematuria. The dose reduction was maintained throughout the treatment and instillation was stopped if toxicity persisted. Intravesical treatment was discontinued if the patient developed febrile neutropaenia (absolute neutrophil count <1000 cells ml^−3^ and temperature⩾38°C), documented bacteraemia in the presence of neutropaenia, grade 3 or 4 neutropaenia/thrombocytopaenia, bilirubin>1.5 × upper limit of normal or transaminase>3 × upper limit of normal. Therapy was delayed by one week in the event of grade 3 bladder toxicity.

### Statistical analysis

For the primary objective of the clinical study, that is, the evaluation of Gem activity, sample size was determined using the optimum Simon's two-stage design (Simon, 1989). ‘Response’ was defined as the lack of residual disease at 6 months, certified by cytological and endoscopic examinations. Positive urinary cytology, new lesions (superficial or infiltrating), nodal involvement and systemic diffusion were considered a ‘no-response’. The study was sized to refuse ‘response’ rates of 40% (*P*_0_) and to provide a significance level of 0.10 with a statistical power of 90% in assessing the activity of the regimen as a 60% ‘response’ rate (*P*_1_). The upper limit for first-stage drug rejection was 11 ‘responses’ in the 28 assessable patients. The upper limit of second-stage rejection was 20 ‘responses’ within a total of 41 assessable patients.

Event-free survival (EFS) was defined as the interval between the date of the first endovesical instillation and the first unfavourable event, superficial disease, progression to infiltrating disease or the last visit. Event-free survival was estimated by the Kaplan–Meier method. Ninety-five percent confidence intervals (95% CI) for ‘response’ and EFS estimates were calculated.

## RESULTS

### *In vitro* studies

**Figure 1:**
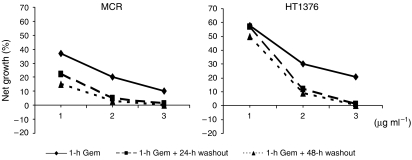
Dose–response cytotoxicity of Gem in bladder cancer cell lines after different treatment schemes.

A cytostatic dose-dependent effect was produced by Gem after all treatment schemes. The effect induced by a 1-h exposure to Gem significantly increased (*P*<0.05) after a 24- and 48-h culture in drug-free medium (wash-out) at all tested concentrations. A generally higher sensitivity of MCR than HT1376 cells was observed (Figure 1). Moreover, the analysis of the types of interaction between the two 1-h exposures to Gem showed an antagonistic effect on cell growth when these were consecutive. Conversely, an additive interaction in HT1376 and synergistic activity in MCR were observed when the two exposures were spaced out by a 24-h (RI=1.5) or 48-h (RI=1.6) wash-out.

Cell cycle perturbations and induction of apoptosis were analysed in an attempt to find an explanation for the different types of interaction. After a 1-h exposure to Gem, we observed a statistically significant increase of cells in G0–G1 phases (67% in treated *vs* 46% in untreated samples, *P*<0.05), together with a similar decrease in both S (25% *vs* 40%, *P*<0.05) and G2–M (7.3% *vs* 14%, *P*<0.05) phases. These perturbations were still present 24 h after Gem removal and the block in G0–G1 phases recovered only after 48 h, after which S phase, the antimetabolite's target, was repopulated (Table 1).

Gem treatment induced apoptosis in about one-third of tumour cells. More specifically, 25% of apoptotic cells were observed after a 1-h exposure to Gem followed by a 72-h culture in drug-free medium, increasing to 35% following the sequential treatment (1-h Gem → 48-h wash-out → 1-h Gem → 24-h wash-out). Conversely, the percentage of necrotic cells never exceeded 5%.

### *In vivo* studies

Forty-one consecutive patients were enrolled onto the study by the Urology Department of Forlì and the Oncology Department of Cattolica. Forty patients completed treatment and were evaluable for clinical end points and one patient was lost to follow-up immediately after registration. All but one of the patients were males, and age ranged from 40 to 87 years (median 66 years). About two-thirds presented a monofocal lesion, 90% had T1 tumours and more than 95% had G2-3 lesions (Table 2).

### Activity

In accordance with a Simon's two-step design, we continued recruitment into the second step, having observed more than 11 responses in the first 28 enrolled patients. On the basis of the primary end point of the study, 38 of the 40 (95%) evaluable patients (95% CI: 83.1–99.4%) obtained a ‘response’ with respect to the minimum expected rate of 60%.

**Figure 2:**
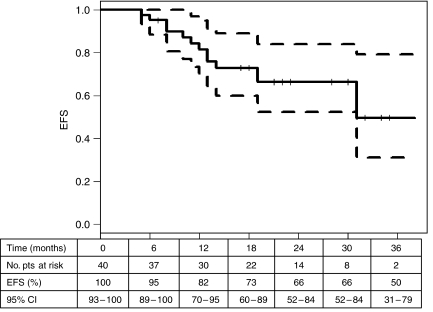
Event-free survival and 95% CI curves.

Specifically, a ‘response’ was observed in 21 of the 23 high-risk tumour patients (91%; 95% CI: 72.0–98.9%) and in all 17 intermediate-risk patients. The probability of event-free survival for the entire case series was around 80% at 1 year and 66% at 2.5 years (Figure 2). At a median follow-up of 28 months, superficial recurrences had been observed in 14 patients. Among these, four relapsed with downstaged (T) disease, three exhibited a lower grade (G) lesion and three had a reduction in both T and G. Finally, two monofocal lesions presented as multifocal at relapse, while the inverse situation was observed in one patient. Only two patients relapsed at 5 and 6 months and both subsequently underwent cystectomy (Table 3).

### Toxicity

Systemic toxicity was very low, with no evidence of alterations in biochemical profiles. Hyperthermia⩾38°C was observed in only one patient and required a Gem dose reduction. Hyperthermia <38°C was observed in six cases and was well controlled by antipyretics. According to NCI-CTC criteria, 10 cases of grade 1 and 27 cases of grade 2 dysuria (pollakiuria and/or stranguria with or without urgency) were observed. Good control of symptoms was obtained in these patients with the administration of anticholinergic drugs. Grade 3 dysuria caused a delay in therapy in only three patients and was resolved with anticholinergics. A consequent reduction in Gem dose was required. Haematuria was never observed.

## DISCUSSION

Although the standard adjuvant treatment for superficial bladder cancer at high-risk of recurrence is intravesical BCG, the role of this therapy is somewhat controversial for intermediate-risk tumours due to the high frequency of side effects and the uncertain risk/benefit ratio. Moreover, there is still no recommended standard therapy for patients relapsing after conventional intravesical treatments. New treatment options are therefore needed, and Gem represents a promising drug.

In a phase I study by Dalbagni *et al* (2002), patients with BCG-refractory bladder carcinoma were treated with biweekly instillations of Gem at scalar doses up to 20 mg ml^−1^ for 6 weeks. This schedule produced a 50% complete remission rate, with acceptable local and systemic toxicity. A lower toxicity was reported by Laufer *et al* (2003) in a dose-finding and pharmacokinetic study using weekly Gem instillations at scalar doses up to 40 mg ml^−1^ for 6 weeks. At the maximum dose, the plasmatic level of Gem was low and grade 2 dysuria was observed in only one patient.

In our clinical protocol, in which the Gem intravesical treatment scheme was derived from preclinical studies designed to evaluate the cytotoxic activity of different Gem schedules, all but one patient concluded the planned treatment. Moreover, moderate urinary toxicity and no haematological side effects were observed, notwithstanding the biweekly instillations, each of 20 mg ml^−1^ of Gem, for 6 weeks.

With regard to Gem activity, some phase II studies (De Berardinis *et al*, 2004; Gontero *et al*, 2004; Campodonico *et al*, 2005) assessed its ablative effect on a single marker lesion left in the bladder after complete resection of all other lesions. In these studies, Gem, administered weekly for 6 weeks, produced a response rate of about 50% with a very low frequency of systemic and local toxicity, which generally did not exceed grade I.

The activity of Gem has also been investigated in BCG-refractory patients following either weekly or biweekly administration. In Bartoletti *et al*'s (2005) study, Gem given once a week for 6 weeks was associated with a 74% and 13% recurrence-free survival in patients with intermediate- and high-risk lesions, respectively, at a median follow up of 13.6 months. The usefulness of the antimetabolite was also confirmed by Kohjimoto in a small series of BCG-refractory patients following biweekly administration (Kohjimoto *et al*, 2005).

In a recent phase II study, 30 BCG-refractory patients were treated biweekly with 2000 mg Gem diluted in 100 ml saline solution for 3 weeks, with each course separated by 1 week's rest (Dalbagni *et al*, 2006). The complete disappearance of all evidence of disease was obtained in 50% of patients and 21% were recurrence-free at 1 year.

In the present study, following a biweekly Gem administration for 6 weeks, 80 and 66% of patients were event-free at the 1- and 2.5-year follow up, respectively, with fairly similar rates in intermediate- and high-risk tumour subgroups. It is also noteworthy that at relapse, lesions were frequently downstaged and only two patients required cystectomy.

These important therapeutic results, which could at least delay the need for invasive cystectomy, were associated with very modest toxicity, underlying the importance of the experimentally derived scheme.

In conclusion, further clinical studies are now needed to verify the efficacy of this Gem schedule in an adjuvant setting, and more preclinical research is warranted to explore and identify the most effective Gem-containing combinations to evaluate in randomised phase II–III studies.

## Figures and Tables

**Table 1:** Distribution of cells in the different cycle phases after exposure to Gem (1 *μ*g ml^−1^)

	**MCR**			**HT1376**		
	**G_0_–G_1_**	**S**	**G_2_–M**	**G_0_–G_1_**	**S**	**G_2_–M**
Untreated cells	46.0±0.7	40.0±0.5	14.0±0.4	50.0±0.6	38.0±0.2	12.0±0.2
Gem (1 h)	67.7^*^±1.5	25.0^*^±1.1	7.3^*^±1.1	70.0^*^±1.2	22.0^*^±1.4	8.0±0.8
Gem (1 h) → wash-out (24 h)	72.5^*^±1.7	20.5^*^±1.2	7.0^*^±1.3	71.8^*^±1.1	19.0^*^±1.1	9.2±0.4
Gem (1 h) → wash-out (48 h)	54.7±0.3	35.5±0.3	9.8±0.2	58.6±1.1	32.0±1.3	9.4±0.3

Data represent mean percentage values±s.d.

^*^P<0.05.

**Table 2:** Patient and tumour characteristics

	***n***	**%**
*Gender*		
Male	38	92.5
Female	2	5.0
		
*Age (years)*		
<60	10	25.0
60–74	17	42.5
⩾75	13	32.5
		
*Focality*		
Monofocal	25	62.5
Mulltifocal	15	37.5
		
*Stage*		
Ta	4	10.0
T1	36	90.0
		
*Grading*		
G1	2	5.0
G2	21	52.5
G3	17	42.5
		
*PS (WHO)*		
0	36	90.0
1	4	10.0

**Table 3:** Variations in Gem-induced pathological characteristics in relapsed patients

		**Focality**		**T**		**G**		
**Patient code**	**Time to recurrence (months)**	**Pre**	**Post**	**Pre**	**Post**	**Pre**	**Post**	**Treatment at relapse**
03	12	M	M	1	1	3	2	Cystectomy (+CIS)
06	13	m	M	1	1	2	2	TURB
07	31	M	m	1	1	2	2	TURB
09	31	m	m	1	a	3	2	TURB
16	10	M	M	1	1	2	2	Cystectomy
19	6	m	M	1	a	2	2	TURB
26	19	M	M	a	a	2	1	TURB
29	19	m	m	a	a	2	2	TURB
30	14	m	m	1	a	3	1	TURB
32	8	m	m	a	a	2	2	TURB
34	13	M	M	1	1	2	2	TURB
37	8	M	M	1	a	2	1	TURB
38	5	M	M	a	a	3	2	TURB
39	11	m	m	1	1	2	2	TURB

T=tumour stage; G=tumour grade; M=multifocal lesion; m=monofocal lesion; CIS=carcinoma *in situ*; TURB=transurethral bladder resection.
